# Analysis of epidemiological characteristics and influencing factors of Long COVID syndrome among university students in the post-pandemic era

**DOI:** 10.3389/fpubh.2026.1771458

**Published:** 2026-08-04

**Authors:** Songqing Guo, Mingma Li, Yuxiang Liu, Yi Zhang, Yuchen Pan, Xinru Wang, Hongqiao Li, Xiangjun Zhai, Xiang Hong, Bei Wang

**Affiliations:** 1 Department of Epidemiology and Health Statistics, School of Public Health, Southeast University, Nanjing, China; 2 Science and Technology Major Project Implementation Office of Jiangsu Provincial Center for Disease Control and Prevention, Nanjing, China

**Keywords:** cohort study, influencing factors analysis, Long COVID syndrome, post-pandemic era, university student population

## Abstract

**Objective:**

To analyze the epidemiological characteristics of Long COVID syndrome in university students during the post-pandemic era, providing evidence for developing prevention strategies and rehabilitation plans for COVID-19 patients.

**Methods:**

A cohort study was conducted in November 2023, recruiting students from a university in Nanjing for baseline investigations. Demographic characteristics, vaccination history, infection history, and venous blood samples were collected to detect SARS-CoV-2 IgG and IgM antibody levels. For individuals with prior COVID-19 infections, Long COVID syndrome status was assessed 1 year post-infection. Multivariate logistic regression analysis was employed to identify influencing factors.

**Results:**

The study included 246 participants (mean age: 21.88 ± 2.06 years; 65.04% female). Median SARS-CoV-2 IgG and IgM levels were 16.500 (105.000, 239.000) AU/ml and 0.095 (0.053, 0.183) AU/ml, respectively. Among 246 participants with prior COVID-19 infection, 82 (33.33%) developed Long COVID syndrome, with alopecia (36.59%), memory decline (28.05%), and sleep disturbances (28.05%) being the most prevalent symptoms. Multivariate analysis revealed that, compared to the non-Long COVID group, depressive symptoms may be positively associated with Long COVID syndrome (adjusted *OR* = 8.235, *95%CI*: 3.478–19.500).

**Conclusion:**

The incidence of Long COVID syndrome among university students is comparable to that of the general population. A bidirectional relationship may exist between depressive symptoms and Long COVID syndrome, warranting increased clinical and public health attention.

## 1 Introduction

On May 5, 2023, the World Health Organization (WHO) officially declared the conclusion of COVID-19 as a global public health emergency (1), signifying humanity’s transition into the post-pandemic era (2, 3). While the pandemic’s acute phase has subsided, its enduring impacts persist through clinical manifestations such as Long COVID syndrome (LCS), which continues to exert substantial long-term health consequences on individual wellbeing and societal functioning (4). Within this context, sustained health surveillance of high-risk populations remains imperative for public health management. University students have emerged as a population of particular research interest due to their distinctive epidemiological characteristics, including an escalating burden of mental health disorders, substantial academic stress, and irregular sleep–wake patterns (5).

Long COVID syndrome has become one of the most pressing public health challenges in the post-pandemic landscape, with its persistent health impairments and associated socioeconomic burdens garnering global attention. Given that university students are undergoing critical developmental stages, the potential ramifications of Long COVID on their cognitive capacities, psychosocial health, and future trajectories warrant rigorous investigation. Understanding the epidemiological profile of Long COVID within this demographic holds significant implications for optimizing healthcare resource allocation, assessing long-term pandemic burdens, and refining post-pandemic public health policies.

Although university students are theoretically considered to be at lower risk of long COVID because of their younger age and the predominantly mild nature of acute infection in this population, they remain an important group for investigation. Existing studies have largely focused on middle-aged and older adults or hospitalized patients, leaving the long-term health consequences in young individuals with mild infection insufficiently characterized. Moreover, persistent symptoms may also occur after mild acute infection. Given the large size of the university student population and their frequent social interactions, long-term health problems in this group may have broad implications for both public health and educational systems. This study therefore aims to conduct a preliminary assessment of the syndrome’s prevalence, clinical presentation spectrum, severity gradients, and associated determinants within this cohort. The findings are expected to provide empirical evidence for developing targeted health intervention strategies in academic institutions.

## 2 Methods

### 2.1 Study population

This study was conducted at two campuses of a university in Nanjing, China. Participants comprised 392 full-time undergraduate, master’s, and doctoral students who voluntarily enrolled through open recruitment. Inclusion and exclusion criteria were established based on research objectives and ethical principles as follows:

Inclusion criteria: (1) Valid student registration at the institution; (2) Full comprehension of study protocols and voluntary provision of written informed consent; (3) Ability to complete baseline surveys, venous blood collection, and scheduled 1-year follow-up.

Exclusion criteria: (1) Current acute infectious diseases of the respiratory system (e.g., seasonal influenza, human metapneumovirus infection); (2) Current acute infectious diseases other than respiratory infections (e.g., infectious diarrhea, varicella) or other severe diseases (e.g., cardiovascular disease, severe anemia); (3) Known immunodeficiency disorders or ongoing systemic immunosuppressive therapy (e.g., post-transplant medications, high-dose glucocorticoids, chemotherapy); (4) Anticipated loss to follow-up during the follow-up period (e.g., graduation, academic leave, withdrawal); (5) New SARS-CoV-2 infection occurring during the follow-up period.

After applying the inclusion and exclusion criteria, a total of 246 participants were ultimately included in the study.

The study protocol was approved by the Ethics Review Committee of Jiangsu Provincial Center for Disease Control and Prevention (no. JSJK2023-B007-02). All participants provided written informed consent prior to enrollment.

### 2.2 Field survey and specimen collection

In November 2023 (baseline survey), participants completed structured questionnaires including:

(1) A self-designed epidemiological survey covering demographic characteristics, behavioral patterns, prior SARS-CoV-2 infection history, and vaccination status;
(2) The 20-item Center for Epidemiologic Studies Depression Scale (CES-D) and 14-item Perceived Stress Scale (PSS-14). CES-D scores range 0–60: <16 (no depressive symptoms), 16–19 (possible depressive symptoms), >19 (depressive symptoms) (6, 7). PSS-14 scores range 0–56: ≤25 (no perceived stress), >25 (perceived stress) (8). Venous blood samples were collected from the median cubital vein. Serum and plasma were separated and stored at −80 °C.

**Figure 1:**
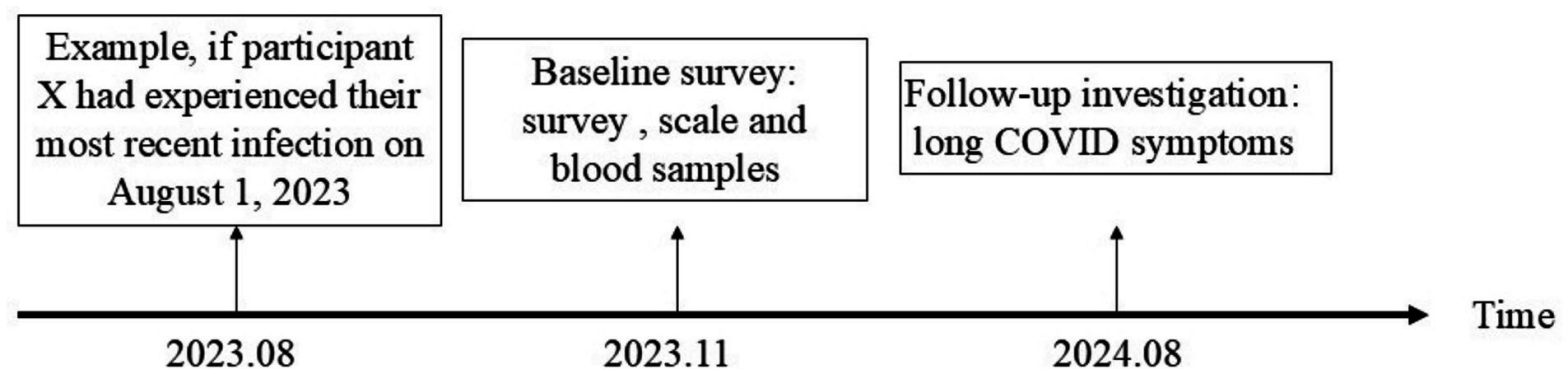
Research timeline.

Given that long COVID symptoms were still ongoing in some participants at the time of the initial survey, a supplementary follow-up investigation was conducted 1 year after infection in order to obtain complete information on the duration of Long COVID syndrome (for example, if the questionnaire survey conducted in November 2023 showed that participant X had experienced their most recent infection on August 1, 2023, a supplementary investigation would be conducted within 1–2 months after August 1, 2024). Research timeline is shown in Figure 1. According to the World Health Organization definition of long COVID, symptoms that occur during the recovery period after SARS-CoV-2 infection and persist for at least 2 months, including fatigue, reduced taste, reduced smell, and memory decline, may affect multiple organ systems and may also lead to cognitive and emotional disturbances (9, 10). In this study, a standardized questionnaire was used to record each symptom. For symptoms that were highly subjective and of low specificity, investigators provided participants with detailed explanations and screening instructions, emphasizing that reported symptoms should represent persistent symptoms following infection rather than discomfort caused by exercise, daily study, work load, or other transient factors (for example, fatigue was reported only when it represented a persistent state following infection, rather than transient fatigue resulting from daily activities). After questionnaire collection, symptom reports with a duration of less than 2 months were excluded.

### 2.3 Laboratory testing

SARS-CoV-2 IgG/IgM antibodies were quantified using the MAGLUMI X8 automated chemiluminescence immunoassay system (Shenzhen New Industries Biomedical Engineering Co., Ltd.) with manufacturer-certified reagents. All procedures strictly adhered to standardized protocols.

### 2.4 Statistical analysis

Data management utilized Microsoft Excel 2021. Normally distributed continuous variables were expressed as mean ± standard deviation (±*s*). Non-normal distribution data were presented as median (interquartile range) [*M* (*P* ~ 25~, *P* ~ 75~)] and analyzed via rank sum tests. Categorical variables were compared using *χ*^2^ tests. When any group had an expected frequency <5, Fisher’s exact test was applied; when the number of groups was large resulting in excessive computational demands for Fisher’s exact test, Monte Carlo simulation was used to estimate the approximate *p* value.

For LCS risk factor analysis, variables with statistical significance in univariate analysis underwent collinearity diagnostics before inclusion in multivariate logistic regression models (reference group: COVID-19 survivors without LCS [non-LCS group = 0]; case group: COVID-19 survivors with LCS [LCS group = 1]). Backward stepwise regression (elimination threshold: *p* > 0.10) generated final models, reporting odds ratios (*OR*) with *95%* confidence intervals (*95%CI*). All analyses employed two-tailed tests (*α* = 0.05) using IBM SPSS Statistics 26.0.

### 2.5 Quality control

Trained investigators conducted standardized data collection. Questionnaire data underwent dual-entry verification by two independent operators, with discrepancies resolved by a third reviewer. Venous blood samples were centrifuged immediately post-collection, aliquoted, and stored at −80 °C to minimize freeze–thaw cycles. Laboratory personnel followed strict SOPs, including pre-experimental validation and quality control measures using certified reference materials.

## 3 Results

### 3.1 Demographic characteristics of study participants

This study enrolled 246 university student volunteers with a mean age of 21.88 ± 2.06 years. The cohort comprised 160 female participants (65.04%). Undergraduate and graduate students accounted for 52.85 and 47.15% of the population, respectively. The CES-D Depression Self-Rating Scale yielded a mean score of 10.68 ± 7.84, with 191 participants (77.64%) classified as asymptomatic, 23 (9.35%) as potentially symptomatic, and 32 (13.01%) as exhibiting depressive symptoms. The PSS-14 Perceived Stress Scale demonstrated a mean score of 34.58 ± 8.02, identifying 225 individuals (91.46%) as non-stressed and 21 (8.54%) as stressed. Serological analysis revealed median SARS-CoV-2 IgG and IgM antibody levels of 16.500 (105.000, 239.000) AU/ml and 0.095 (0.053, 0.183) AU/ml, respectively.

### 3.2 Prevalence of Long COVID syndrome

**Figure 2:**
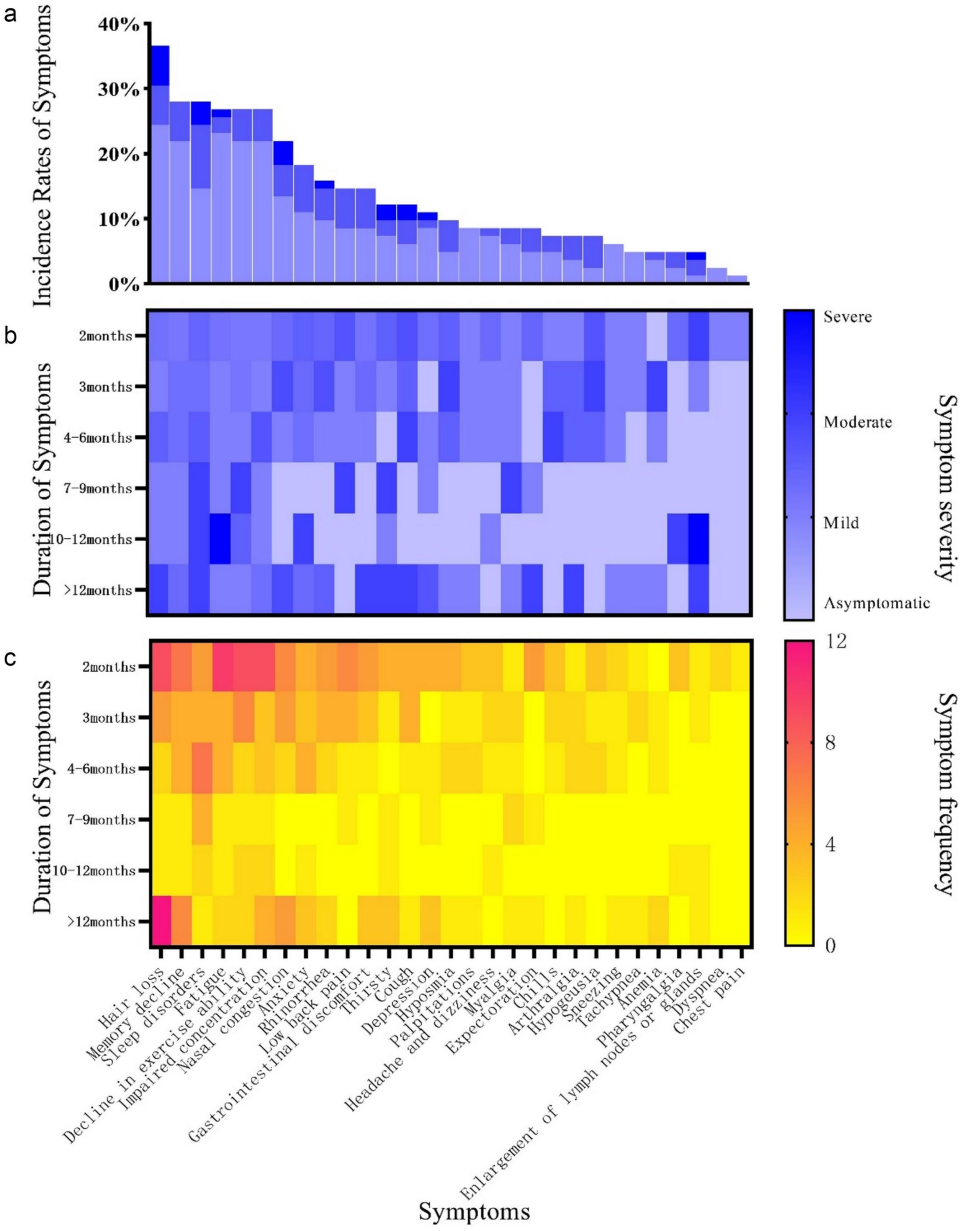
Temporal persistence and severity stratification of Long COVID syndrome manifestations in 82 affected individuals. (**a**) The lower heatmap employs a gradient color scheme to delineate the frequency distribution of symptom duration across various long COVID manifestations. (**b**) The central heatmap utilizes chromatic intensity to represent the gradation of symptom severity throughout their persistence period. (**c**) The upper stacked bar chart maintains chromatic consistency with the central heatmap, illustrating the proportional distribution of mild, moderate, and severe symptom categories within each clinical manifestation. Mild: Symptoms are present but have essentially no significant impact on daily academic performance and routine life. Moderate: Symptoms have caused a certain degree of impairment in learning efficiency, sleep quality, or daily activities, yet the individual is still able to maintain normal academic and daily life. Severe: Symptoms significantly interfere with academic pursuits, social interactions, or daily activities, necessitating rest, reduced activity levels, or medical intervention.

Follow-up investigations of Long COVID syndrome showed that among 246 participants with a history of prior COVID-19 infection, 82 (33.33% [27.44, 39.22%]) exhibited at least one Long COVID symptom; of these, 25 reported only one persistent symptom, whereas the remaining 57 reported two or more persistent symptoms. The most prevalent manifestations were alopecia (36.59%), memory decline (28.05%), and sleep disturbances (28.05%), with alopecia and sleep disturbances showing the highest proportions of moderate and severe cases at 16.67%/16.67 and 34.78%/13.04%, respectively. Because one participant may have experienced more than one long COVID symptom, the total number of symptoms exceeded the total number of individuals with Long COVID syndrome. Details are shown in Figure 2.

### 3.3 Determinants of Long COVID syndrome

#### 3.3.1 Univariate analysis

Participants were stratified into control and Long COVID groups for epidemiological characterization and univariate analysis. Non-parametric tests were applied to non-normally distributed antibody data, while chi-square tests assessed other factors. Statistically significant differences emerged in depressive symptoms (*χ*^2^ = 28.867, *p* < 0.001) and perceived stress (*χ*^2^ = 8.434, *p* = 0.004). Details are shown in Table 1.

**Table 1:** Univariate analysis of factors associated with Long COVID syndrome.

Variable	Non-long COVID group (%)	Long COVID group (%)	*χ*^2^/Fisher/*Z*^c^	*p*
Gender			0.009	0.925
Female	107 (65.24)	53 (64.63)		
Male	57 (34.76)	29 (35.37)		
BMI			–	0.644
<18.5	23 (14.02)	9 (10.98)		
18.5–23.9	106 (64.63)	59 (71.95)		
24.0–27.9	26 (15.85)	9 (10.98)		
≥28.0	9 (5.49)	5 (6.10)		
Student population			0.131	0.718
Undergraduate	88 (53.66)	42 (51.22)		
Graduate	76 (46.34)	40 (48.78)		
Alcohol consumption			–	0.425
Never	116 (70.73)	53 (64.63)		
Occasional	44 (26.83)	25 (30.49)		
Frequent	4 (2.44)	4 (4.88)		
Smoking status			–	0.667
Non-smoker	160 (97.56)	81 (98.78)		
Former smoker	4 (2.44)	1 (1.22)		
Mask-wearing frequency			0.110	0.991
Never	14 (8.54)	7 (8.54)		
Occasional	103 (62.80)	53 (64.63)		
Regular	20 (12.20)	9 (10.98)		
Frequent	27 (16.46)	13 (15.85)		
Hand hygiene			–	0.768
Poor	3 (1.83)	3 (3.66)		
Fair	55 (33.54)	26 (31.71)		
Good	87 (53.05)	42 (51.22)		
Excellent	19 (11.59)	11 (13.41)		
Weekly outings			0.399	0.819
1–2 times	93 (56.71)	45 (54.88)		
3–4 times	48 (29.27)	23 (28.05)		
≥5 times	23 (14.02)	14 (17.07)		
Exercise frequency			2.063	0.559
Never	9 (5.49)	7 (8.54)		
Occasional	90 (54.88)	39 (47.56)		
Regular	28 (17.07)	13 (15.85)		
Frequent	37 (22.56)	23 (28.05)		
Time since last infection			0.325	0.568
Period A^b^	106 (64.63)	56 (68.29)		
Period B	58 (35.37)	26 (31.71)		
Severity of last infection			1.079	0.299
Mild	150	78		
Moderate	14	4		
Time since last vaccination			–	0.416
Unvaccinated	8 (4.88)	3 (3.66)		
>12 months	144 (87.80)	69 (84.15)		
≤12 months	12 (7.32)	10 (12.20)		
Vaccination doses			–	0.511
Unvaccinated	8 (4.88)	3 (3.66)		
1–2 doses	28 (17.07)	19 (23.17)		
3–4 doses	128 (78.05)	60 (73.17)		
Vaccination regimen			–	0.914
Unvaccinated	8 (4.88)	3 (3.66)		
Homologous vaccination	140 (85.37)	70 (85.37)		
Heterologous (sequential) vaccination	16 (9.76)	9 (10.98)		
CES-D Depression scale			28.867	<0.001
No depressive symptoms	140 (85.37)	51 (62.20)		
Possible depressive symptoms	16 (9.76)	7 (8.54)		
Depressive symptoms	8 (4.88)	24 (29.27)		
PSS-14 stress scale			8.434	0.004
No perceived stress	156 (95.12)	69 (84.15)		
Perceived stress	8 (4.88)	13 (15.85)		
IgG^a^	163.500 (102.250, 239.000)	172.500 (118.500, 247.500)	7484.000	0.149
IgM	0.100 (0.055, 0.186)	0.083 (0.053, 0.182)	6350.500	0.478
Total	164 (100.00)	82 (100.00)		

^a^IgG and IgM measurements were subjected to logarithmic transformation prior to statistical analysis, with results expressed as median values (25th percentile, 75th percentile). ^b^Period A (December 1, 2022 to January 31, 2023) refers to the high-transmission phase after the relaxation of COVID-19 control measures in China, during which the number of infections increased sharply; Period B (February 1, 2023 to the baseline survey) refers to the later phase, during which the number of infections declined substantially, although sporadic infections still occurred. ^c^*χ*^2^/Fisher/*Z* column indicates that Fisher’s exact test was applied.

#### 3.3.2 Multivariate logistic regression analysis

Variables and coding schemes for regression modeling are detailed in Table 2.

**Table 2:** Variable names and coding.

Variable	Coding
CES-D Depression scale	0 = No depressive symptoms, 1 = Possible depressive symptoms, 2 = Depressive symptoms
PSS-14 stress scale	0 = No perceived stress, 1 = Perceived stress

The two factors demonstrating statistical significance in univariate analysis were included in multivariate logistic regression analysis (all *VIF* <5 for collinearity diagnostics). The perceived stress factor (*p* = 0.756) was excluded from the final model. The final results showed that individuals assessed as having depressive symptoms by the CES-D Depression Self-Rating Scale were highly associated with Long COVID syndrome (*p* < 0.001), with an *OR (95%CI)* of 8.235 (3.478, 19.500) (Table 3). (This study also attempted to incorporate common parameters that could theoretically influence the outcome, such as vaccination and infection characteristics (which showed no statistical significance in univariate analysis), into the multivariate analysis, and all such models yielded the same results as above.)

**Table 3:** Multivariate logistic regression analysis of factors associated with Long COVID syndrome.

Factor (reference)	Variable	Long COVID group	
		*OR* (*95%CI*)	*p*
CES-D Depression scale (no depressive symptoms)	Possible depressive symptoms	1.021 (0.467, 3.088)	0.704
	Depressive symptoms	8.235 (3.478, 19.500)	<0.001

## 4 Discussion

In the post-pandemic era, the persistent public health implications of SARS-CoV-2 infection remain evident. However, epidemiological investigations into Long COVID syndrome among university students—a distinct demographic group—remain insufficient. This study aims to preliminarily characterize LCS manifestations in this population through a volunteer-based investigation. Our findings demonstrate an LCS incidence rate of 33.33%, aligning with the 10–30% range reported in expert consensus documents and the 29.62% prevalence identified in Wang et al.’s meta-analysis (11, 12), albeit with divergent symptom profiles. While prior studies by Qin et al. and Li et al. identified fatigue, memory impairment, and reduced exercise capacity as predominant manifestations (13, 14), our cohort exhibited distinct prevalence patterns characterized by alopecia, memory decline, and sleep disturbances. These discrepancies may reflect age-dependent variations in LCS symptomatology. The substantial proportion of severe alopecia and sleep disturbances observed in our cohort suggests potential associations with psychological comorbidities (15), while pre-existing subclinical conditions exacerbated by infection warrant consideration.

Logistic regression analysis revealed a significant positive correlation between CES-D-assessed depressive symptoms and LCS occurrence, implying that post-infection depression may serve as a relevant factor for LCS development. This association could be mediated by compromised immune regulation in individuals with depressive tendencies, thereby elevating LCS susceptibility. Supporting this hypothesis, Zachary et al. documented reduced LCS incidence among depression patients receiving psychotropic interventions (16). Intriguingly, self-reported depression attributed to LCS constituted less than half of scale-identified cases, suggesting either under recognition of depressive states or bidirectional causality wherein LCS induces depressive symptomatology. This aligns with existing literature positing that chronic LCS manifestations may precipitate neuropsychiatric sequelae (17, 18). Collectively, our findings propose a potential bidirectional relationship between depression and LCS, necessitating further mechanistic validation.

Conducted during the pandemic transition phase, this year-long cohort study systematically characterizes LCS epidemiology and relevant factors in university populations, providing evidence for tailored campus health strategies. Limitations include unexamined nonspecific symptoms in uninfected controls, precluding comparative analyses, and indicating the need for improvement in future studies; undetermined IgG-LCS causality requiring longitudinal verification through repeated-measures analyses in subsequent cohort studies; potential recall bias in self-reported data and although our investigators provided detailed explanations of the questionnaire items to improve participants’ understanding, information bias and possible misclassification due to certain nonspecific symptoms could not be completely avoided; restricted generalizability due to limited sample size and single-institution sampling, such that the findings may be primarily applicable to university students with mild acute infection, while the small number of participants with moderate acute infection limited further assessment of the influence of acute disease severity on Long COVID syndrome; and absence of viral genotyping or extended follow-up. Future investigations should incorporate multicenter designs, expanded cohorts, and serial biomarker assessments.

## 5 Conclusion

In conclusion, this study identifies comparable LCS prevalence between university students and general populations, with alopecia, memory decline, and sleep disturbances constituting the principal clinical manifestations of Long COVID syndrome. The findings suggest a potential bidirectional association between depressive symptoms and Long COVID syndrome among infected individuals, warranting increased attention. We advocate that universities prioritize and sustain surveillance of long COVID health burdens among student populations, integrating such efforts into post-pandemic campus public health management systems and health support programs.

## Data Availability

The datasets presented in this article are not readily available because due to the nature of this research, participants of this study did not agree for their data to be shared publicly, so supporting data is not available. Requests to access the datasets should be directed to 382163395@qq.com.
